# How to Manage Self-Poisoning With Baclofen in Alcohol Use Disorder? Current Updates

**DOI:** 10.3389/fpsyt.2018.00417

**Published:** 2018-09-04

**Authors:** Nicolas Franchitto, Benjamin Rolland, Fanny Pelissier, Nicolas Simon

**Affiliations:** ^1^Department of Addiction Medicine, Toulouse-Purpan University Hospital, Toulouse, France; ^2^Poison Control Center, Toulouse-Purpan University Hospital, Toulouse, France; ^3^UMR 1027, Paul Sabatier University, Toulouse, France; ^4^Service Universitaire d'Addictologie de Lyon (SUAL), Pôle MOPHA, CH Le Vinatier, Bron, France; ^5^Univ Lyon, Inserm U1028, CNRS UMR5292, UCBL, CRNL, Lyon, France; ^6^APHM, INSERM, IRD, SESSTIM, Hop Sainte Marguerite, Service de Pharmacologie Clinique, CAP-TV, Aix Marseille Univ, Marseille, France

**Keywords:** baclofen, intoxication/pharmacology, comorbid conditions, seizures, psychiatry

## Abstract

Specialists in addiction medicine continue to debate whether baclofen is still indicated to treat alcohol use disorders in view of conflicting results as to its efficacy. This review summarizes current knowledge on self-poisoning with baclofen focusing of alcohol-use disorder in order to provide an overview of the reliable scientific knowledge on management of such an intoxication. Moreover, as alcohol-dependent patients experience many psychiatric co-morbidities, the risk in suicide attempt using baclofen seems real. Numerous studies have suggested that patients given daily-doses of baclofen higher than 80 mg/day are more likely to attempt suicides than others. Following an ingestion of a large amount of baclofen, central nervous system depression is usually observed. Seizures require the patient to be admitted in intensive care unit and should be treated like other toxicological seizures. Cardiac complications include prolonged QTc interval, degree heart block, premature atrial contractions, and supraventricular tachycardia, hypotension and bradycardia. In cases of intoxication, the elimination half-life of baclofen may last between 12 and 36 h post-overdose and renal failure is known to delay its clearance. Rarely measured in clinical practice, the toxic level of baclofen blood level ranges from 1.1 to 3.5 mg/l, and coma or fatal intoxication are observed from 6 to 9.6 mg/l. Baclofen withdrawal has been observed but making the diagnosis of withdrawal in case of suspected self-poisoning is difficult as baclofen intoxication and baclofen withdrawal share many clinical signs. Admission to hospital to manage of suicide attempt with baclofen is mandatory and should not be limited to baclofen alone. It needs to include other aspects of the overall care of patients with alcohol disorders (psychological and psychosocial interventions, management of comorbid mental conditions and physical complications).

## Introduction

Baclofen has been commonly used to treat spasticity in neurological diseases because of its muscle relaxant effects. As it acts on gamma-aminobutyric acid (GABA)-B receptors in the spinal cord, it is used to treat muscle spasm associated with spinal cord diseases and injury. Since 2002, many medical teams have studied the use of baclofen in the management of alcohol-dependent patients. Conflicting results were observed in double-blind randomized clinical trials (RCTs) conducted to assess the efficacy of baclofen at both low doses, i.e., between 30 and 50 mg/d (1–3) and at higher doses, up to 400 mg/d (4). Nonetheless, recent RCTs failed to show that baclofen is superior in terms of long-term follow-up with such high doses (5–7). Baclofen was found to be safe and well-tolerated in alcohol-dependent patients including those suffering from liver cirrhosis (2). Nonetheless, guidelines or evidence are still lacking about how to prescribe baclofen in this indication. Meanwhile, baclofen is increasingly prescribed at both low and high doses, especially in European countries. The increasing number of prescriptions has been followed by an increasing number of cases of self-poisoning with baclofen. In France, since 2008, the use of high-dose baclofen (HDB), that is, up to 300 mg/day and sometimes more, has rapidly spread among general practitioners and alcohol addiction specialists. This has led to discussions between specialists, particularly with regard to increased risk of suicide attempts.

The increasingly widespread use of baclofen leads to greater likelihood of intentional and unintentional exposures. Evidence in the literature suggests that the effects of baclofen overdose are usually severe and require admission to intensive care. Seizures, cardiac arrest and fatal outcomes have been reported. In view of this conflicting information, we performed a systematic review of the literature on baclofen overdose, focusing on patients with alcohol use disorder (AUD). We aimed to present an overview of the manifestations of acute baclofen overdose, to determine whether serum concentrations are predictive of poisoning severity, and to describe the effectiveness of the therapeutic interventions used to manage an overdose.

## Background: brief overview of the sensitive context of baclofen prescription

Specialists in addiction medicine continue to debate whether baclofen is still indicated to treat AUD in view of conflicting results as to its efficacy. In many countries, baclofen is prescribed off-label. In France, baclofen may be prescribed under temporary recommendation for use (TRU). In July 2017, the French National Agency for the Safety of Medicines and Health Products (ANSM) revised the TRU asking physicians not to give baclofen to alcohol-dependent patients at a daily dose higher than 80 mg/d. The optimal daily dose of baclofen for AUD has not yet been approved, but it may be prescribed to alcohol-dependent patients whose goal is not to reach abstinence but to reduce consumption and when approved drugs have failed (8).

In the literature, the daily dose given to patients varied depending on the objectives of the study. Some authors prescribed low to moderate doses, while others used high doses. As chronic baclofen consumption is considered to induce tolerance, it is important to bear this in mind.

## Overview of self-poisoning with baclofen reported by numerous antipoison centers

Intoxication with baclofen has been described for decades. Since the growing interest in its use to treat AUD, numerous studies have focused on self-poisoning in alcohol-dependent patients (9–14). In 2006, Leung et al. (9) showed that self-poisoning with baclofen was responsible for severe symptoms when the supposed ingested dose (SID) was higher than 200 mg. However, the authors gave no details on comorbid AUD or current treatment with baclofen at the time of intoxication.

Studies performed by poison centers showed that the SID at the time of self-poisoning was 400 mg (15) and 480.7 mg (10), higher than the dose believed to cause severe intoxication.

A recent study performed by the French National Safety Agency for Medicines and Health Products (Agence Nationale de Sécurité des Médicaments et des produits de santé, ANSM) assumed that among all causes of death in baclofen-treated patients (*n* = 385), 97 were believed to be the result of a suicide attempt. The number of suicides with baclofen differed according to the daily dose taken: 9 suicides occurred in patients receiving a dose lower than 30 mg/day, 10 in patients receiving 30 to 75 mg/day, 6 in patients receiving 75 to 180 mg/day, and 2 in patients receiving doses higher than 180 mg/day. Such an observation in this study is of paramount importance, as it might be thought that patients given high doses of baclofen were more likely to attempt suicide than those given lower doses under 80 mg/day. Baclofen at a high dose is believed to reduce anxiety (16) and increase sedation (17), which may induce disinhibition at the time of peak drug effect, triggering suicide attempt in alcohol-dependent patients over-represented among suicide victims. Conversely, suicide attempt because of behavioral disinhibition related to hypomanic episodes induced by baclofen given at high dose (18) have been described (19, 20). Nonetheless, constructive conclusions cannot be reached without taking a holistic view of the patient (21).

## Clinical presentation

Clinical features described in self-poisoning with baclofen are summarized in Table 1. Following acute ingestion of a large amount of baclofen, central nervous system depression ranging from drowsiness to coma is usually observed. The threshold leading to CNS depression has been arbitrarily defined by Leung et al. (9) as a dose higher than 200 mg. However, the mg/kg dose or the patient's weight were not given.

**Table 1:** Clinical features described in self-poisoning with baclofen.

	**Clinical features**	**References**
Central nervous system findings	Decreased level of consciousness (drowsiness to coma)	(9, 26)
	Seizures	(10, 12, 14, 15)
	Burst suppression	(23, 24)
Laboratory findings	Acidosis	(24)
	Rhabdomyolysis	(25)
Cardiovascular findings	Prolonged QTc interval	(25)
	First-degree heart block	(26)
	Premature atrial contractions	(13)
	Supraventricular tachycardia	(12, 27)
	Bradycardia	(13, 14)
Pulmonary findings	Respiratory depression	(14, 27)
	Aspiration pneumonia	(10, 15)

Seizures are usually reported in baclofen overdose, requiring the patient's admission to an intensive care unit (6, 7, 12, 14–16). The pathophysiological mechanisms of these seizures are still poorly understood. Baclofen is known to be a proconvulsant drug, mediated by complex GABA-B regulation of both the GABAergic and glutamatergic systems. Experimental data suggest that activation of GABA-B receptors could accentuate neural excitation contrast in some parts of the brain (22).

As baclofen is usually co-ingested with alcohol and other medications, the seizure threshold is decreased and the patient should be closely monitored. Baclofen-induced seizures should be treated like other toxicological seizures. Benzodiazepines are the first-line treatment but should be associated with propofol and barbiturates if needed. Seizure activity is evidenced by continuous electroencephalogram monitoring.

Baclofen overdose is also responsible for encephalopathy, with a clinical presentation typical for brain-death (23). Marked electroencephalographic abnormalities combine slowing down of the background activity with paroxysmal activity. In severe overdose, burst-suppression has been observed (24), and electroencephalogram should be continuously recorded until the abnormal features disappear.

Metabolic acidosis although not a common finding, has also been reported. It should be seen as complications of decreased consciousness or coma, co-ingested drugs or rhabdomyolysis.

The cardiac complications include prolonged QTc interval (25), first-degree heart block (26), premature atrial contractions (13) and supraventricular tachycardia (12, 27), which are related to autonomic nervous system disturbances. In view of the similarities of baclofen to GABA, it may decrease the sympathetic outflow leading to hypotension and bradycardia. Autonomic nervous system disturbances may also result in respiratory depression, although in baclofen overdose a central respiratory effect cannot be ruled out.

## The pharmacokinetics of intoxication

Many studies have focused on changes in the half-life of baclofen, as this may influence the management of intoxication. At a therapeutic dose, its half-life is about 2–6 h with a reported average of 3.5 h. However, in alcohol-dependent patients in particular, wide individual variations have been observed, showing linear pharmacokinetics with a proportional relationship only with doses between 30 and 240 mg per day (28).

In cases of intoxication, the elimination half-life of baclofen may be as long as 12 to 36 h post-overdose (29). Patients need to be closely monitored because of prolonged sedation and coma and the increased risk of seizures.

## Renal replacement therapy, intoxication with baclofen and renal function

Renal failure is known to delay clearance of baclofen and an accumulation phenomenon can occur (30), exposing patients to complications. In patients with impaired renal function, the drug is almost completely eliminated from the body by renal filtration and tubular secretion, whereas only a minor pathway involves liver metabolism (31). The value of hemodialysis after overdose is still under debate. When the patient is severely intoxicated, some authors recommend a renal replacement therapy, in particular continuous veno-venous hemofiltration to increase baclofen elimination, even if renal function is normal (32). They argue that renal replacement therapy is indicated as baclofen is moderately lipophilic and is moderately bound to protein, with a limited volume of distribution (~1 L/kg). Conversely, other authors have underlined the fact that in patients whose renal function is normal, extracorporeal treatments including hemodialysis and hemofiltration do not enhance the elimination of baclofen (33). Other authors reported a case of a 29-year-old woman who took 3500 mg of baclofen (31). Hemodialysis sessions were guided according to baclofen plasma concentrations. A delayed rebound in baclofen plasma concentration was associated with recurrence of toxicity after each hemodialysis session. The authors stated that the cause was either sustained retention of baclofen in tissue or red cells or was due to continuing absorption from a pharmacobezoar. Although pharmacobezoar is a rare event, Cleophax et al. observed the resolution of intoxication after the late introduction of charcoal and poly-ethylene-glycol, following the repeated failure of hemodialysis. They concluded that baclofen elimination was not enhanced by hemodialysis in this patient with normal renal function and that gastro-intestinal decontamination should be performed if delay rebound in plasma baclofen concentration is observed (31).

## Plasma baclofen concentration

Blood baclofen levels are rarely measured in clinical practice, unless intoxication is considered as severe (10, 15, 25, 31) or if the patient has renal impairment. The toxic level ranges from 1.1 to 3.5 mg/l and coma or fatal intoxication are observed from 6 to 9.6 mg/l (34). Conversely, the therapeutic level is <0.6 mg/l. Nonetheless, whatever the plasma concentration, baclofen penetrates the blood-brain barrier. Although its concentration in the brain is lower than in serum, it is eliminated more slowly from the central nervous system than from serum. This observation is of paramount importance as CNS depression may persist even after plasma baclofen levels return to normal (29).

## Baclofen withdrawal

Baclofen withdrawal has been observed after abrupt discontinuation of the drug (35). When a patient with suspected self-poisoning with baclofen is admitted to an emergency department, withdrawal is difficult to diagnose as baclofen intoxication and withdrawal share many clinical signs: worsening spasticity, muscle spasms, status epilepticus, hallucinations, pruritus, hyperthermia, rhabdomyolysis and multisystem organ failure, in some cases leading to death.

## Discussion

The most frequent cause of severe intoxication with baclofen is self-poisoning. Accidental exposures have been described, with less severe clinical features (10). Acute recreational intoxication with baclofen has occurred leading to coma (26). Focusing on AUD, because alcohol-dependent patients experience psychiatric comorbidity there is a real risk of self-poisoning. In many patients, early symptoms were followed by delayed worsening of their condition which required rapid admission to an emergency department or intensive care unit. When baclofen self-poisoning occurs at home, because of its potential severity transportation by emergency medical services, with close monitoring of clinical status and vital signs, should be considered. The rapidity of the deterioration may be difficult to assess, especially in the pre-hospital setting, firstly because the onset of symptoms may be abrupt depending on the co-intoxicants (10), and secondly because the interval between the poisoning and the emergency call is usually not known, particularly if the patient is unconscious.

Because suicidal ideation and suicide attempts are more prevalent in people with substance use disorders than in the general population, admission to hospital is mandatory particularly after a suicide attempt. The patient must be seen by a psychiatrist after being detoxified (36).

The risk of coma should be stressed. CNS depression is a well-recognized symptom associated with baclofen intoxication and is increased by concomitant ethanol consumption. It should be kept in mind that baclofen may be taken at the same time as alcohol by patients for whom total abstinence is not the goal (8, 37). Sedation, dizziness and confusion are by far the most frequent symptoms related to self-poisoning with baclofen. Baclofen-induced seizures should be treated like other toxicological seizures. Benzodiazepines are the first-line treatment, and propofol and barbiturates should be used for refractory seizures. Monitoring the blood level of baclofen at the same time as continuous electroencephalogram monitoring may be indicated. Nonetheless, the blood level of baclofen does not correlate with coma duration as the drug penetrates the blood-brain barrier and is eliminated more slowly from the central nervous system (CNS) than from serum. This leads to prolonged CNS depression even after plasma baclofen levels return to normal (29). The efficacy of gastric decontamination in baclofen overdose patients has not been proven. In one case of massive intoxication with baclofen (SID 3500 mg), the attending physician administered polyethylene glycol and activated charcoal on day 9 after the self-poisoning, which resulted in decreased plasma baclofen concentration (31).

Patients who experience bradycardia after baclofen overdose should be given an atropine bolus to reverse central vagal stimulation induced by baclofen (13).

Patients should be monitored for significant morbidity (e.g., aspiration). In patients with severe intoxication and/or elevated creatinine kinase level as a complication of coma or hypotension, renal function should be monitored by serum measurement of urea nitrogen and creatinine. Intravenous hydration may be required to prevent renal failure. Serial monitoring of serum creatinine kinase activity may help in gauging the efficacy of treatment interventions and help the physician start renal replacement therapy.

No antidote exists. Empirical administration of flumazenil to reverse coma is not advisable. Because polydrug intoxication including alcohol with baclofen is commonplace, flumazenil may trigger seizure although this complication is rare.

From the literature, it appears that 200 mg is the ingested dose at which moderate to severe symptoms can be expected to occur (9). However, the authors give no details on any current treatment with baclofen, which limits the interpretation of these results. Moreover, the absorption of higher doses, particularly in suicide attempts, has since been described by poison centers.

An important point that needs further investigation, and which may explain the high doses of baclofen taken with the purpose of self-harm, is the development of tolerance during prolonged treatment. This underlines the need to assess the daily dose taken by the patient (21, 38) at the time of the suicide attempt. Tolerance may lead to variability in the clinical features of intoxication and even decrease its severity. Although development of tolerance to baclofen attenuates baclofen-induced encephalopathy in rats (24), further investigations are warranted. Tolerance to baclofen may affect patient management by differentiating between acutely intoxicated patients with no previous treatment by baclofen and baclofen-treated patients (“acute-on-chronic poisoning”).

Patients with acute-on-chronic poisoning require particular attention as they are at risk of baclofen withdrawal. The treating physicians should not neglect to reintroduce baclofen, although the dose that should be given is still debated. Titrated doses should be the rule.

Management should not be limited to baclofen alone. It needs to include other aspects of the overall care of patients with alcohol disorders (psychological and psychosocial interventions, management of comorbid mental conditions and physical complications) (38).

## Author contributions

NF and FP conceived the paper and performed literature search. NF, FP, BR, and NS wrote the paper. All authors critically reviewed the manuscript and agreed on its final version.

### Conflict of interest statement

The authors declare that the research was conducted in the absence of any commercial or financial relationships that could be construed as a potential conflict of interest.
